# Supplementary material for: Parasites and RNA viruses in wild and laboratory reared bumble bees Bombus pauloensis (Hymenoptera: Apidae) from Uruguay
Source: PLoS One. 2021 Apr 26;16(4):e0249842. doi: 10.1371/journal.pone.0249842 (PMC8075198; doi:10.1371/journal.pone.0249842)
Supplement: S1 Table — (PDF) [file pone.0249842.s001.pdf]

Table 1. Presence of phoretic mites

Groups	Phoretic mites				
	T. putrescentiae (number of ind)	*P. longanalis (number of ind)*	*P. longuipilus (number of ind)*	*Kuzinia (number of ind)*	*P. fucorum (number of ind)*
Lab workers					
Lab workers					
Lab workers	2				
Lab workers	9		1		
Lab workers	9		1		
Lab workers	24		1		
Lab workers	41				
Lab workers	3				
Lab workers					
Lab workers	7				
Lab workers	8				
Lab workers	1				
Lab workers					
Lab workers	1				
Lab workers					
Lab workers					
Lab workers					
Lab workers					
Lab workers					
Lab workers					
Lab workers					
Lab workers				1	
Lab workers	10				
Lab workers				1	
Lab workers	3				
Lab workers				1	
Lab workers	5				
Lab workers	5				
Lab workers	10				
Lab workers				2	
Lab workers	3			1	
Lab workers					
Lab workers	8				
Lab workers	5				
Lab workers	3				
Lab workers					
Lab workers	7		1		

Lab workers	2				
Lab workers					
Lab workers	2				
Lab workers					
Lab workers	1				
Lab workers	1			2	
Lab workers	2				
Lab workers	9				
Lab workers					
Wild queens					
Wild queens	7				
Wild queens	33		1		
Wild queens	101				
Wild queens	1				
Wild queens	10		1		
Wild queens					
Wild queens					
Wild queens					
Wild queens		37	2	1	
Wild queens	1				
Wild queens	51	3	1	1	
Wild queens	40	9			
Wild queens	106	1		1	
Wild queens	6	1			
Wild queens	64	1		2	
Wild queens					
Wild queens	84		1		
wild workers					
wild workers					
wild workers					
wild workers	1		1		
wild workers					
wild workers					
wild workers					
wild workers					
wild workers					
wild workers					
wild workers					
wild workers					
wild workers					
wild workers	2				
wild workers					
wild workers					
wild workers					
wild workers					

wild workers	2				
wild workers					
wild workers					
wild workers					
wild workers					
wild workers	2				
wild workers					
wild workers					
wild workers				1	
wild workers				25	
wild workers				7	
wild workers				6	
wild workers				15	
wild workers				22	
wild workers				2	
wild workers				17	
wild workers				18	
wild workers		4		1	1
wild workers				20	
Wild queens					
Wild queens	7				
Wild queens	33		1		
Wild queens	101				
Wild queens	1				
Wild queens	10		1		
Wild queens					
Wild queens					
Wild queens					
Wild queens		37	2	1	
Wild queens	1				
Wild queens	51	3	1	1	
Wild queens	40	9			
Wild queens	106	1		1	
Wild queens	6	1			
Wild queens	64	1		2	
Wild queens					
Wild queens	84		1		

Table 2. Presence of internal parasites

	Internal parasites			
Groups	*N. ceranae*	*T. pampeana*	*S. bombi*	Diptera
wild workers	1			

wild workers				
wild workers				
wild workers				
wild workers	1			
wild workers				
wild workers				
wild workers				
wild workers				
wild workers		1		
wild workers				
wild workers				
wild workers				
wild workers				
wild workers				
wild workers				
wild workers				
wild workers		1		
wild workers		1		
wild workers		1		
wild workers		1		
wild workers	1			
wild workers	1			
wild workers	1			
wild workers				
wild workers				
wild workers				
wild workers				
wild workers				
wild workers				
wild workers				
wild workers				
wild workers	1			
wild workers				
wild workers		1		
wild workers				
wild workers				
wild workers				
wild workers				
wild workers				
wild workers				
wild workers				
wild workers				
wild workers		1		
wild workers				
wild workers				
Wild queens				
Wild queens				

Wild queens				
Wild queens			1	
Wild queens		1		
Wild queens				
Wild queens			1	
Wild queens				
Wild queens				
Wild queens	1			
Wild queens		1		
Wild queens		1		
Wild queens				
Wild queens				
Wild queens	1	1		
Wild queens				
Wild queens				
Wild queens	1			
Wild workers	1			
Wild workers	1			
Wild workers				
Wild workers	1			
Wild workers	1			
Wild workers	1			
Wild workers				
Wild workers	1			
Wild workers				
Wild workers	1			
Wild workers				1
Wild workers				
Wild workers				1
Wild workers				
Wild workers	1			
Wild workers	1	1		
Wild workers	1			
Wild workers	1			
Wild workers	1			
Wild workers		1		
Wild workers				
Wild workers	1	1		
Wild workers				
Wild workers				
Wild workers	1			
Wild workers	1			
Wild workers				
Wild workers				
Wild workers				
Wild workers				

Wild workers				
Wild workers				
Wild workers	1			
Wild workers				
Wild workers				
Wild workers	1			
Wild workers				

Table 3. Presences of RNA viruses

	VIRUS ARN			
Group	BQCV	DWV	ABPV	SBV
Lab workers	1			
Lab workers	1			
Lab workers	1		1	
Lab workers	1			
Lab workers	1			
Lab workers	1			
Lab workers	1			
Lab workers	1		1	1
Lab workers	1			
Lab workers	1			
Lab workers	1			
Lab workers	1			
Lab workers	1			
Lab workers	1			
Lab workers	1			
Lab workers	1			1
Lab workers	1			
Lab workers	1			
Lab workers	1			
Lab workers	1			
Lab workers				
Lab workers	1			
Lab workers	1			
Lab workers	1			
Lab workers	1			
Lab workers	1			
Lab workers	1		1	
Lab workers	1		1	
Lab workers	1			1
Lab workers	1			
Lab workers	1			
Lab workers	1			
Lab workers	1			

Lab workers	1			1
Lab workers	1			
Lab workers	1			
Lab workers	1			
Lab workers	1			
Lab workers	1		1	
Lab workers	1			
Lab workers	1			
Wild queens	1		1	1
Wild queens	1		1	1
Wild queens	1			
Wild queens	1			
Wild queens	1		1	
Wild queens	1			
Wild queens	1			
Wild queens	1			
Wild queens	1			
Wild queens	1			
Wild queens	1		1	
Wild queens	1			1
Wild workers	1			
Wild workers	1			
Wild workers	1			
Wild workers	1			
Wild workers	1	1		
Wild workers	1			
Wild workers	1	1		
Wild workers	1	1		
Wild workers	1		1	
Wild workers	1			
Wild workers	1			
Wild workers	1	1		
Wild workers	1		1	
Wild workers	1		1	
Wild workers	1			
Wild workers	1			
