# Supplementary material for: Parasites and RNA viruses in wild and laboratory reared bumble bees Bombus pauloensis (Hymenoptera: Apidae) from Uruguay
Source: PLoS One. 2021 Apr 26;16(4):e0249842. doi: 10.1371/journal.pone.0249842 (PMC8075198; doi:10.1371/journal.pone.0249842)
Supplement: S2 Table — (PDF) [file pone.0249842.s002.pdf]

Group	Mite species				Number of species	Number of mites	D	Simpson (1-D)	Diversity categories
	Kuzinia	Tyrophagus	P. longanalis	Parasitellus					
Lab worker	0	2	0	0	1	2	1,00	0,00	Low
Lab worker	0	9	0	0	1	9	1,00	0,00	Low
Lab worker	0	9	0	0	1	9	1,00	0,00	Low
Lab worker	0	24	0	0	1	24	1,00	0,00	Low
Lab worker	0	41	0	0	1	41	1,00	0,00	Low
Lab worker	0	3	0	0	1	3	1,00	0,00	Low
Lab worker	0	7	0	0	1	7	1,00	0,00	Low
Lab worker	0	8	0	0	1	8	1,00	0,00	Low
Lab worker	0	1	0	0	1	1	1,00	0,00	Low
Lab worker	0	1	0	0	1	1	1,00	0,00	Low
Lab worker	1	0	0	0	1	1	1,00	0,00	Low
Lab worker	0	10	0	0	1	10	1,00	0,00	Low
Lab worker	1	0	0	0	1	1	1,00	0,00	Low
Lab worker	0	3	0	0	1	3	1,00	0,00	Low
Lab worker	1	0	0	0	1	1	1,00	0,00	Low
Lab worker	0	5	0	0	1	5	1,00	0,00	Low
Lab worker	0	5	0	0	1	5	1,00	0,00	Low
Lab worker	0	10	0	0	1	10	1,00	0,00	Low
Lab worker	2	0	0	0	1	2	1,00	0,00	Low
Lab worker	1	3	0	0	2	4	0,50	0,50	Moderate
Lab worker	0	8	0	0	1	8	1,00	0,00	Low
Lab worker	0	5	0	0	1	5	1,00	0,00	Low
Lab worker	0	3	0	0	1	3	1,00	0,00	Low
Lab worker	0	7	0	0	1	7	1,00	0,00	Low
Lab worker	0	2	0	0	1	2	1,00	0,00	Low
Lab worker	0	2	0	0	1	2	1,00	0,00	Low
Lab worker	0	1	0	0	1	1	1,00	0,00	Low
Lab worker	2	1	0	0	2	3	0,33	0,67	High
Lab worker	0	2	0	0	1	2	1,00	0,00	Low
Lab worker	0	9	0	0	1	9	1,00	0,00	Low
Wild queens	0	7	0	0	1	7	1,00	0,00	Low
Wild queens	0	33	0	0	1	33	1,00	0,00	Low
Wild queens	0	101	0	0	1	101	1,00	0,00	Low
Wild queens	0	1	0	0	1	1	1,00	0,00	Low
Wild queens	0	10	0	0	1	10	1,00	0,00	Low
Wild queens	1	0	37	0	2	38	0,95	0,05	Low
Wild queens	0	1	0	0	1	1	1,00	0,00	Low
Wild queens	1	51	3	0	3	55	0,86	0,14	Low
Wild queens	0	40	9	0	2	49	0,69	0,31	Moderate
Wild queens	1	106	1	0	3	108	0,96	0,04	Low
Wild queens	0	6	1	0	2	7	0,71	0,29	Low
Wild queens	2	64	1	0	3	67	0,91	0,09	Low
Wild queens	0	13	0	0	1	13	1,00	0,00	Low
Wild queens	0	84	0	0	1	84	1,00	0,00	Low
Wild workers	0	1	0	0	1	1	1,00	0,00	Low

Wild workers	0	2	0	0	1	2	1,00	0,00	Low
Wild workers	0	2	0	0	1	2	1,00	0,00	Low
Wild workers	1	0	0	0	1	1	1,00	0,00	Low
Wild workers	25	0	0	0	1	25	1,00	0,00	Low
Wild workers	7	0	0	0	1	7	1,00	0,00	Low
Wild workers	6	0	0	0	1	6	1,00	0,00	Low
Wild workers	15	0	0	0	1	15	1,00	0,00	Low
Wild workers	22	0	0	0	1	22	1,00	0,00	Low
Wild workers	2	0	0	0	1	2	1,00	0,00	Low
Wild workers	17	0	0	0	1	17	1,00	0,00	Low
Wild workers	18	0	0	0	1	18	1,00	0,00	Low
Wild workers	1	0	4	1	3	6	0,40	0,60	High
Wild workers	20	0	0	0	1	20	1,00	0,00	Low
