# Supplementary material for: Parasites and RNA viruses in wild and laboratory reared bumble bees Bombus pauloensis (Hymenoptera: Apidae) from Uruguay
Source: PLoS One. 2021 Apr 26;16(4):e0249842. doi: 10.1371/journal.pone.0249842 (PMC8075198; doi:10.1371/journal.pone.0249842)

PLOS ONE

Parasites and RNA viruses in wild and laboratory reared bumblebees Bombus pauloensis (Hymenoptera: Apidae) from Uruguay

--Manuscript Draft--

**Manuscript Number:**	PONE-D-20-36603
**Article Type:**	Research Article
**Full Title:**	Parasites and RNA viruses in wild and laboratory reared bumblebees Bombus pauloensis (Hymenoptera: Apidae) from Uruguay
**Short Title:**	Parasites and RNa viruses in wild and laboratory reared bumblebees in Uruguay
**Corresponding Author:**	Sheena Marie Salvarrey, PhD<br>Universidad de la República Uruguay Facultad de Ciencias<br>Montevideo, Montevideo URUGUAY
**Keywords:**	Bombus spp.; mites; Microsporidia; viruses; spillover
**Abstract:**	Bumblebees (Bombus spp.) are important pollinators for the maintenance of natural ecosystems and food production. Bombus pauloensis is a widely distributed species in South America that recently began to be commercialized. In this study, B. pauloensis was screened for the presence of mites, internal parasites, and RNA viruses in laboratory rearing workers, wild workers, and queens collected after hibernation. The screening included the foretic mites Tyrophagus putrescentiae, Pneumolaelaps longanalis, Pneumolaelaps longipilus, Kuzinia spp. and Parasitellus fucorum , the microsporidia Nosema ceranae and Tubulinosema pampeana , a dipteran of the family Conopidae , the nematode Sphaerularia bombi and Black queen cell virus (BQCV), Deformed wing virus (DWV), Acute paralysis virus (ABCV) and Sacbrood virus (SBV). Of the different groups analyzed, the queens were the ones with the highest number of mite species and it was the only group in which the presence of Conopidae and nematodes of the species S. bombi was detected. In the case of microsporidia, a higher prevalence of N. ceranae was detected in field workers. Finally, the bumblebees presented the four RNA viruses studied for A. mellifera, in proportions similar to those previously reported in this species. Those results highlights the risks of spillover among the different species of pollinators.
**Order of Authors:**	Sheena Marie Salvarrey, PhD
	Karina Antúnez
	Daniela Arredondo
	Santiago Plischuk
	Pablo Revainera
	Matías Maggi
	Ciro Invernizzi
**Additional Information:**	
**Question**	**Response**
**Financial Disclosure**<br><br>Enter a financial disclosure statement that describes the sources of funding for the work included in this submission. Review the submission guidelines for detailed requirements. View published research articles from *PLOS ONE* for specific examples.	No. The author(s) received no specific funding for this work

Powered by Editorial Manager® and ProduXion Manager® from Aries Systems Corporation

This statement is required for submission and **will appear in the published article** if the submission is accepted. Please make sure it is accurate.<br><br>**Unfunded studies**<br>Enter: *The author(s) received no specific funding for this work.*<br><br>**Funded studies**<br>Enter a statement with the following details:<br>• Initials of the authors who received each award<br>• Grant numbers awarded to each author<br>• The full name of each funder<br>• URL of each funder website<br>• Did the sponsors or funders play any role in the study design, data collection and analysis, decision to publish, or preparation of the manuscript?<br>• **NO** - Include this sentence at the end of your statement: *The funders had no role in study design, data collection and analysis, decision to publish, or preparation of the manuscript.*<br>• **YES** - Specify the role(s) played.<br><br>* typeset	
**Competing Interests**<br><br>Use the instructions below to enter a competing interest statement for this submission. On behalf of all authors, disclose any competing interests that could be perceived to bias this work—acknowledging all financial support and any other relevant financial or non-financial competing interests.<br><br>This statement **will appear in the published article** if the submission is accepted. Please make sure it is accurate. View published research articles from *PLOS ONE* for specific examples.	The authors have declared that no competing interests exist.

**NO authors have competing interests**<br><br>Enter: *The authors have declared that no competing interests exist.*<br><br>**Authors with competing interests**<br><br>Enter competing interest details beginning with this statement:<br><br>*I have read the journal's policy and the authors of this manuscript have the following competing interests: [insert competing interests here]*<br><br>* typeset	
**Ethics Statement**<br><br>Enter an ethics statement for this submission. This statement is required if the study involved:<br><br>• Human participants<br>• Human specimens or tissue<br>• Vertebrate animals or cephalopods<br>• Vertebrate embryos or tissues<br>• Field research<br><br>Write "N/A" if the submission does not require an ethics statement.<br><br>General guidance is provided below. Consult the submission guidelines for detailed instructions. **Make sure that all information entered here is included in the Methods section of the manuscript.**	N/A

**Format for specific study types**<br><br>**Human Subject Research (involving human participants and/or tissue)**<br>• Give the name of the institutional review board or ethics committee that approved the study<br>• Include the approval number and/or a statement indicating approval of this research<br>• Indicate the form of consent obtained (written/oral) or the reason that consent was not obtained (e.g. the data were analyzed anonymously)<br><br>**Animal Research (involving vertebrate animals, embryos or tissues)**<br>• Provide the name of the Institutional Animal Care and Use Committee (IACUC) or other relevant ethics board that reviewed the study protocol, and indicate whether they approved this research or granted a formal waiver of ethical approval<br>• Include an approval number if one was obtained<br>• If the study involved *non-human primates*, add *additional details* about animal welfare and steps taken to ameliorate suffering<br>• If anesthesia, euthanasia, or any kind of animal sacrifice is part of the study, include briefly which substances and/or methods were applied<br><br>**Field Research**<br><br>Include the following details if this study involves the collection of plant, animal, or other materials from a natural setting:<br>• Field permit number<br>• Name of the institution or relevant body that granted permission	
**Data Availability**<br><br>Authors are required to make all data underlying the findings described fully available, without restriction, and from the time of publication. PLOS allows rare exceptions to address legal and ethical concerns. See the PLOS Data Policy and FAQ for detailed information.	Yes - all data are fully available without restriction

A Data Availability Statement describing where the data can be found is required at submission. Your answers to this question constitute the Data Availability Statement and **will be published in the article**, if accepted.<br><br>**Important:** Stating 'data available on request from the author' is not sufficient. If your data are only available upon request, select 'No' for the first question and explain your exceptional situation in the text box.<br><br>Do the authors confirm that all data underlying the findings described in their manuscript are fully available without restriction?	
**Describe where the data may be found in full sentences. If you are copying our sample text, replace any instances of XXX with the appropriate details.**<br><br>• If the data are **held or will be held in a public repository**, include URLs, accession numbers or DOIs. If this information will only be available after acceptance, indicate this by ticking the box below. For example: *All XXX files are available from the XXX database (accession number(s) XXX, XXX.).*<br>• If the data are all contained **within the manuscript and/or Supporting Information files**, enter the following: *All relevant data are within the manuscript and its Supporting Information files.*<br>• If neither of these applies but you are able to provide **details of access elsewhere**, with or without limitations, please do so. For example:<br><br>*Data cannot be shared publicly because of [XXX]. Data are available from the XXX Institutional Data Access / Ethics Committee (contact via XXX) for researchers who meet the criteria for access to confidential data.*<br><br>*The data underlying the results presented in the study are available from (include the name of the third party*	All relevant data are within the manuscript and its Supporting Information files.

and contact information or URL).<br>• This text is appropriate if the data are owned by a third party and authors do not have permission to share the data.<br><br>* typeset	
Additional data availability information:	Tick here if your circumstances are not covered by the questions above and you need the journal’s help to make your data available.

Manuscript Click here to access/download;Manuscript;Manuscript Salvarrey et al.doc

Parasites and RNA viruses in wild and laboratory reared bumblebees *Bombus pauloensis* (Hymenoptera: Apidae) from Uruguay

Sheena Salvarrey[1*], Karina Antúnez[2], Daniela Arredondo[2], Santiago Plischuk[3], Pablo Revainera[4], Matías Maggi[4], Ciro Invernizzi[1]

[1]Sección Etología, Facultad de Ciencias, Montevideo, Uruguay.
[2]Departamento de Microbiología, Instituto de Investigaciones Biológicas Clemente Estable (IIBCE), Montevideo, Uruguay.
[3]Centro de Estudios Parasitológicos y de Vectores (CEPAVE) (CONICET-UNLP) La Plata, Argentina.
[4]Centro de Investigación en Abejas Sociales (CIAS), Facultad de Ciencias Exactas y Naturales, Mar del Plata, Argentina.

Corresponding autor: ssalvarrey@fcien.edu.uy
Iguá 4225, CP 14000. Montevideo, Uruguay

Abstract

Bumblebees (*Bombus* spp.) are important pollinators for the maintenance of natural ecosystems and food production. *Bombus pauloensis* is a widely distributed species in South America that recently began to be commercialized. In this study, *B. pauloensis* was screened for the presence of mites, internal parasites, and RNA viruses in laboratory rearing workers, wild workers, and queens collected after hibernation. The screening included the foretic mites *Tyrophagus putrescentiae, Pneumolaelaps longanalis, Pneumolaelaps longipilus, Kuzinia* spp. and *Parasitellus fucorum*, the microsporidia *Nosema ceranae* and *Tubulinosema pampeana*, a dipteran of the family *Conopidae*, the nematode *Sphaerularia bombi* and Black queen cell virus (BQCV), Deformed wing virus (DWV), Acute paralysis virus (ABCV) and Sacbrood virus (SBV). Of the different groups analyzed, the queens were the ones with the highest number of mite species and it was the only group in which the presence of Conopidae and nematodes of the species *S. bombi* was detected. In the case of microsporidia, a higher prevalence of *N. ceranae* was detected in field workers. Finally, the bumblebees presented the four RNA viruses studied for *A. mellifera,* in proportions similar to those previously reported in this species. Those results highlights the risks of spillover among the different species of pollinators.

Key words: *Bombus* spp., mites, Microsporidia, viruses, spillover

Introduction

Wild and managed pollinators are essential for agricultural production, maintenance of biodiversity and the sustainability of natural ecosystems [1–3]. However, they are threatened by different factors including intensification of land use, intoxication with pesticides or infection by multiple pest and pathogens, between others [2].

In particular, wild bumble bees populations of the genus *Bombus* (Hymenoptera: Apidae), are in global decline [4,5]. Among the main threats for bumble bee health, different parasitic enemies stand out, some of them are specific to the genus *Bombus*, while others have a broad host spectrum [6,7]. The extended commerce and movement of honey bees *Apis mellifera* has led to the dispersion of pathogens to infect new hosts, a phenomenon known as spillover [6,8–10].

The microsporidium *Nosema ceranae* [recently Tokarev *et al.* (2020)[11] suggest to be reclassified as *Vairimorpha ceranae*)] is one of the most documented spillover examples. This parasite is found in honey bees [12], bumblebees [8,13–16], stingless bees [17,18], solitary bees (Euglossini) [19] and social wasps [18]. Another example of pathogen spillover are RNA viruses. Acute bee paralysis virus (ABPV), Black queen cell virus (BQCV), Deformed wing virus (DWV) and Sacbrood virus (SBV) [20,21] were described in honeybees but has also been found in bumblebees [9,22,23], stingless bees [24,25], carpenter bees [26] and other insects such as syrphids (Diptera) [27] and butterflies (Lepidoptera) [28].

Bombus pauloensis (=*Bombus atratus*) is widely distributed throughout South America [29,30], and is utilized successfully in production of tomato (*Solanum lycopersicum* L.)

and pepper (*Capsicum annum* L.) in greenhouses [31–33], as well as that of red clover (*Trifolium pratense* L.) seeds [34]. The colonies of *B. pauloensis* have been raised in captivity in small scale both in Colombia and Uruguay [35,36] and at commercial scale in Argentina, following a very extended breeding practice of some European and North American species [37,38]. From a sanitary point of view, the artificial breeding conditions (high density of individuals, impossibility of going out to defecate and forage, and limited food availability), can increase the survival and multiplication of different pathogens, facilitating the proliferation and transmission of diseases [39,40].

Bombus pauloensis is distributed throughout the Uruguayan territory, and alongside *Bombus bellicosus*, whose distribution is more reduced, are the only two *Bombus* species found in the country [41]. Previous studies reported the presence of internal and external parasites in queens, workers and males of both species [15,42,43]. The list of internal parasites includes the microsporidia *N. ceranae* and *Tubulinosema pampeana*, the nematode *Sphaerularia bombi* and at least one species of parasitoid diptera [42]. The external mites, *Kuzinia* spp., *Pneumolaelaps longanalis*, *Pneumolaelaps longipilus*, *Scutacarus acarorum,* and *Tyrophagus putrescentiae* were also found and reported by Revainera *et al.* [43].

In the southern region of South America (Argentina and Chile) the dispersion of the exotic species *Bombus terrestris* and *Bombus ruderatus*, has put under threat native bumblebees species, as *Bombus dahlbomii*, which is currently endangered [44–47]. These invasive species, introduced in Chile over the last few years, may have been acting as reservoirs of pathogens that jumped to native species causing significate damage [44,47]. Uruguay, as a neighbor country of Argentina, is not free of the invasion

of *B. terrestris* or *B. ruderatus*, or even new pathogens present on these species and dispersed by others.

In this study we perform exhaustive survey of the external and internal parasites as well as RNA viruses associated to queens and wild workers of *B. pauloensis* collected in field conditions, as in workers from colonies obtained by artificial rearing.

Materials and methods

Bumblebee collection

During the spring of 2014, 33 queens of *B. pauloensis* were collected in the Faculty of Agronomy, University of the Republic, Montevideo (34º 50' S, 56º 13' W) after finishing their hibernation period. Among these, 19 queens were used for parasite analysis, 14 for viral analysis and 40 to start laboratory rearing according to Salvarrey *et al.* [36]. At the end of the season, 92 workers reared under lab conditions were collected. Among these, 46 were used for parasite analysis and 46 for viral analysis.

In February, 2015, 54 wild workers were collected in the Faculty of Agronomy, from which 37 were used for parasite analysis and 17 for virus analysis. The individuals used for parasite analysis were kept at -20 °C, and those assigned to virus analysis were kept at -80°C.

Identification of mites and internal parasites.

In order to detect phoretic mites, individual bumblebees were observed with a magnifying glass (40x), and the extract mites and were separated, clarified observed with a light microscope (400X) and identified using taxonomic keys [48–51].

Prevalence (percentage of bumblebee with mites), abundance (number of mites per examined bumblebee), and intensity (number of mites per parasitized bumblebee) was determined for each mite species and in the three bumblebees groups (queens, wild and laboratory workers).

Besides that, mite diversity per group was calculated using Simpson's index. Furthermore, taking into account those values and following the criterion suggested by Revainera *et al.* (2019)[43], three categories were build: low diversity (0-0.3), moderate (0.3-0.6) and high (0.6-1).

For internal parasite identification, bumblebees were dissected under a stereoscopic microscope (10x – 40x). Firstly, the metasomal cavity was thoroughly observed looking for nematodes and diptera larvae, and trachea was scutinized in search of mites [42]. Then, small samples of fat tissue, Malphigian tubules, midgut and posterior intestine were extracted and observed under compound bright field microscope (400x - 1000x) in order to detect microsporidia and protists [52]. Special attention was given to the fat tissue since the abnormal presence of granules in this tissue could be provoked by the presence of *T. pampeana* [53]. In the cases in which microsporidia was observed, the body of the infected insects was completely homogenized using 2 ml of distilled water and the number of microsporidia spores was quantified using a Neubauer chamber [54].

Analyses of RNA viruses

Workers and queens samples were individually placed in 1.5 ml tubes and 500 µl or 1200 µl of PBS, respectively, were added. Individuals were disrupted and homogenized using a sterile glass rod. Total RNA was isolated from each individual bee using the PureLink® Viral RNA/DNA Mini Kit (Invitrogen™). Co-purified DNA was degraded usingDNase I, Amplification Grade (Invitrogen™), according to the manufacturer´s recommendations. Then the reverse transcription to cDNA was performed using the High Capacity cDNA Reverse Transcription Kit (Applied Biosystems™, EEUU), according to the manufacturer´s instructions. Viral detection was carried out by real time PCR using Power SYBR® Green PCR Master Mix (Applied Biosystems, EEUU) and specific *primers* for reference and viral genes (Table 1). Reaction mixture consisted of 1X Master Mix, 0.5 µM of each *primer*, RNAse free water and 5 µl of 1:10 diluted cDNA in a final volume of 25 µl. Negative controls were included on each run. Serial dilutions of a mix of all the samples were used as a standard curve.

Table 1. Primers utilized for the quantification of viruses in the samples through qPCR.

Primer	Sequence 5' – 3'	Virus/Gen	Reference
ABPV1	ACCGACAAAGGGTATGATGC	ABPV	Johnson *et al*., 2009
ABPV2	CTTGAGTTTGCGGTGTTCCT		
DWV_F	CTGTATGTGGTGTGCCTGGT	DWV	Kukielka *et al*., 2008
DWV_R	TTCAAACAATCCGTGAATATAGTGT		
BQCV_F	AAGGGTGTGGATTTCGTCAG	BQCV	Kukielka *et al.,* 2008
BQCV_R	GGCGTACCGATAAAGATGGA		
SBV_F	GGGTCGAGTGGTACTGGAAA	SBV	Johnson *et al*., 2009
SBV_R	ACACAACACTCGTGGGTGAC		

BACTIN1	ATGCCAACACTGTCCTTTCTGG	β-actina	Yang & Cox-Foster, 2005
BACTIN2	GACCCACCAATCCATACGGA		

Real time PCR reactions were carried out in a thermal Bio-Rad CFX96 Touch ™ Real-Time System (Bio-Rad, USA). The cycling program consisted of an initial activation at 95 ºC for 10 minutes, and 40 cycles of 95 ºC for 15 seconds, 50 ºC for 30 seconds and 60 ºC for 30 seconds.

The specificity of the reaction was verified through the inclusion of a *melting* curve the amplified products (from 65 to 95 ºC The expression ratio between each target gene and the geometric mean of references genes (RPS 5 and β-actin) was calculated according to the method described by Pfaffl [55].

Statistical analyses

The prevalence of the different pathogens and mites in the queens, wild and laboratory workers was compared using the Chi-square test. The intensity of the infection by microsporidia as well as the number of mites in the three groups of bumblebees mentioned were compared using the Kruskal Wallis test and Mann-Whitney. P values under 0.05 were considered significant.

Results

Multiple parasites and pathogens were identified on bumble bees, including the mites *T. putrescentiae*, *P. longanalis*, *P. longipilus*, *Kuzinia* sp. and *Parasitellus fucorum*, the microsporidia *N. ceranae* and *T. pampeana*, a diptera of Conopidae family, the nematode *S. bombi,* and the RNA viruses BQCV, ABPV, SBV and DWV.

Phoretic mites

Fifty eight percent of the screened bumblebees were infected by at least one species of mite. The prevalence was higher in the queens (73.6%), followed by the laboratory workers (65.2%) and the wild workers (40.5%) (χ^2= 7.52; $P< 0.05$). The mite more frequently found were *T. putrescentiae*, which was detected mainly in queens and laboratory workers (H= 21.56; $P< 0.05$) and *Kuzinia* sp. in the wild workers (H= 15.36; $P< 0.05$) (Table 2).

Table 2. Prevalence (P), abundance (A) and intensity (I) of the observed mites on laboratory workers, wild workers and queens of *B. pauloensis.*

Bumblebee group		***T. putrescentiae***	***P. longanalis***	***P. longipilus***	***Kuzinia* spp.**	***P. fucorum***
Laboratory workers	P	**58.7**	-	8.7	13.0	-
	A	4.0	-	0.0	0.2	-
	I	6.7	-	1.0	1.3	-
Wild workers	P	10.8	2.7	2.7	**29.7**	2.7

	A	0.2	0.1	0.0	3.6	0.0
	I	1.8	4.0	1.0	1.3	1.0
Queens (laboratory)	P	**63.2**	**31.6**	26.3	21.1	-
	A	26.5	2.7	0.3	0.3	-
	I	42.0	8.7	1.2	1.3	-
Total	P	42.2	6.9	9.8	20.6	1.0
	A	6.8	0.5	0.1	1.4	0.0
	I	16.1	8.0	1.1	7.0	1.0

Queens were infested by the highest number of mites species (χ^2 = 12.89; $P< 0.05$) and 64.3% of them presented between two and four species of mites per individual. This co-infection was less observed in wild workers or in laboratory workers (13.3% and 16.6%, respectively).

The mean Simpsons' diversity index calculated for mites was 0.08 for the lab worker bees, 0.12 for the wild workers and 0.18 for the queens. The classification for the bumblebees of each group showed that over 90% had a low diversity of mites (Fig 1).

Fig 1. Diversity of phoretic mites in bumblebees. Proportion of laboratory workers, wild workers and queens of *B. pauloensis* that based on the values of Simpson's diversity index presented a low (0-0.3), moderate (0.3-0.6) and high (0.6-1) diversity of phoretic mites following the criterion of Revainera *et al.* [43].

Internal pathogens

The microsporidia *N. ceranae* and *T. pampeana* were found in the three analyzed groups of bumblebees. The prevalence of *N. ceranae* taking into account all samples was 26.5%. Significant differences were found in the prevalence between groups (χ^2= 14.72; $P< 0.05$), being higher in the wild workers (45.9%) than the laboratory workers (13%) (χ^2 = 12.6; $P< 0.05$) and the queens (16.6%) (χ^2 = 5.78; P= 0.02). The prevalence values of the last two groups were similar (χ^2= 0.08; P= 0.77). Regarding the intensity of the infections, similar values were found in the three groups (*U*= 1.58; P= 0.45). Queens showed 2.1 ± 2.9 x 10^6 spores/~~individual~~, wild workers 2.4 ± 0.96 x 10^5 spores/~~individual~~ and the laboratory workers 3.4 ± 4.8 x 10^5 spores/~~individual~~.

Almost fourteen percent of the bumblebees were infected with *T. pampeana* (21% of the queens, 8.1% of the wild workers, 15.2% of the laboratory workers). No significant differences were found in the prevalence per groups (χ^2 = 1.93; P= 0.38). Regarding the intensity of the infection, queens showed 6.4 ± 2.5 x 10^5 spores/~~individual~~, wild workers 5.2 ± 8.0 x 10^5 spores/~~individual~~ and laboratory workers 2.4 ± 3.5 x 10^5 spores/individual, with no differences between the groups (*U*= 2.86; P= 0.23).

Three cases of coinfection (2.9%) with both types of microsporidia were found, two in wild workers and one in a queen.

Parasites

The nematode *S. bombi* was found in two of the 19 analyzed queens (10.5%), counting a total of seven gravid females (hypertrophied uteri) in one of them, and two in the other.

Parasitoids

Diptera larvae belonging to Conopidae family were found in six wild workers (16.2%, n= 37) and in two queens (10.5%, n=19) (χ^2 = 7.69; P< 0.05). Any parasitoid was found in laboratory workers.

RNA Virus

In 83.8% of the analyzed bumblebees at least one RNA virus was detected. The BQCV was the most prevalent virus (80.9% of the samples), while SBV, DWV and ABPV showed lower values (Fig 2). Regarding the detection of virus among the analyzed groups, differences were found for the DWV (χ^2 = 13.8; P< 0.05) and for the SBV (χ^2 = 10.69; P< 0.05), since those were more prevalent in wild workers and queens, respectively (Fig 2).

Fig 2. Prevalence of the virus BQCV, DWV, ABPV and SBV in laboratory workers, wild workers and queens. The * indicates significate differences (P < 0.05) between the bumblebee groups for the Chi-square test.

Of the analyzed bumblebees 55.8% were only infected by one virus, mainly BQCV; while 27.9% showed co-infection with different viruses, including BQCV-ABPV (n = 9), BQCV-DWV (n=4), BQCV-SBV (n = 3) and ABPV-SBV (n=1). Triple infection was found in two samples, with only one case found in laboratory workers and queens.

Discussion

Bumblebee colonies have an annual life cycle and only the queens survive the winter. This factor has shaped the behavior of parasites and pathogens to reproduce and spread beyond the period in which colonies disappear [56–58].

In this study we evaluated the presence of the main internal and external parasites, as well as the RNA viruses on queens wild and laboratory workers of *B. pauloensis.* Results allowed the analysis of the effect of the caste and breeding conditions in the prevalence of different parasitosis and virosis.

Phoretic mites

The effect of mites in bumblebee populations is unclear. Many groups feed on wax and pollen, while others consume small nematodes and fungi, which might be beneficial for bumblebees [59–61]. However, mites can act as vectors facilitating the introduction of fungi and pathogens. In this sense, Revainera *et al.* [62] found in individuals of *P. longanalis* *P. fucorum* obtained from bumblebees collected since 1940’s, the presence of *Ascosphaera* spp., *N. ceranae*, *Nosema apis*, and *Nosema bombi*, *Crithidia bombi*, *Lotmaria passim* (Euglonozoa; Trypanosomatidae), *Apicystis bombi* (Apicomplexa:

Neogregarinorida), and *A. mellifera* filamentous virus (AmFV), highlighting the importance that these mites have in the transmission of diseases and raising doubts about the propagation routes of some parasites. Furthermore, in their phoretic stage mites can also affect the flight ability and therefore affect the foraging behavior of the individuals [63].

Out of the five mite species found in this study, four (*T. putrescentiae*, *P. longanalis*, *P. longipilus*, *Kuzinia* sp.) had already been reported to be associated to *B. pauloensis* in Uruguay [43]. In this case, besides the species mentioned, the presence of *P. fucorum* was noted in one wild bumblebee worker. On the other hand, *S. acarorum* was not found, maybe due to the reduced values of prevalence and intensity in the country [43].

The queens showed the highest number of mite species, which is reasonable since they are the only individuals in the colony that survive and make it through the winter with mites attached to their bodies [57,64,65]. Even so, Simpson's Diversity Index showed low diversity values on the mites of the three groups.

The laboratory workers and queens showed a high number of *T. putrescentiae* individuals, which is know for its cosmopolitan distribution and its preference for high fat and/or protein contain food. The nest boxes used bumblebees breeding in captivity offer an unbeatable place for this mite proliferation since nests provide an abundant amount of pollen and wax, rich in protein and fat. Additionally, the confinement increases the lack of hygiene, which makes it difficult to control the presence of this mite, situation that has been reported in the laboratory breeding of other insects [51].

The mites of the genus *Kuzinia* were associated to wild workers and queens. These feed exclusively on pollen, so they can find their food both in and out of the bumblebee

nest, which would explain their abundance in the individuals that were foraging in the fields and their scarcity in those bumblebees that were confined to a nest [66,67]. Their presence in queens is expectable since these were collected fresh out of their hibernation and it is in this stage that the bumblebee cycle begins promoting the phoresis of the mites. Their phoretical habits are associated with bees, wasps, beetles and other groups of insects [68]. The taxonomy of this group is in revision due to the controversies in the determination of species. According to some taxonomists, there are three species: *K. affinis, K. laevis and K. americana*. However ever when their identification is based in morphology (body size and shape, and number of setae in the tarsiI-IV) that are still unclear [50,68].

The two species of *Pneumolaelaps*, feed directly on pollen and wax from the nests, gathering near the larvae to receive the food. Even when feeding this way, mites are heavily associated to queens [69], which matches with the results showed in this study. Meanwhile*, P. fucorum,* is a mite of great size that feeds on pollen and small arthropods present on the bumblebee nest [65,66]. In this study a single specimen was found, in accordance with recent studies in where a low prevalence or even absence of this mite was noted [43,59,61].

~~Pathogens~~

The microsporidia *N. ceranae* and *T. pampeana* were found in the three groups of bumblebees. In Uruguay both species had already been associated to *B. pauloensis* [15,42]. The natural host of *N. ceranae* is the Asian bee *Apis cerana* [70], ~~however~~, it

was found infecting many species of bumblebees around the world, which could impact negatively on their populations [8,23,71,72].

Nosema ceranae showed higher prevalence in wild workers (45.9%) than in lab workers or queens. This prevalence was different than previous studies in which a prevalence of 72% was reported in workers collected in 2010 [15] and 28.6% in 2012 [42].

Even though that in queens the prevalence of *N. ceranae* was a third of what the wild workers showed, the spore count was approximately ten times bigger than the one exhibited by the two groups of workers. The lack of statistical back up in the level of infection in the three groups, me be associated to the low number of individuals affected (only two queens). These results do not match with those found by Plischuk *et al*. [42] in *B. pauloensis* from Uruguay, where workers were more infected than queens.

The differences in the prevalence and infection level found between different studies could be due regional differences and time of the year in which bumblebees were collected, as in the sampling effort. In honey bees, the prevalence of *N. ceranae* varies within the region and time of the year [73,74]. Evenmore, the pollen diversity available for honeybees also influence the infection level [75,76]. This point has been barely studied in bumblebees. Rotheray *et al*. [77] found in *B. terrestris*, that there is a negative relationship between *N. ceranae* infection level and the amount of food (pollen and sugar syrup) that was given to the colonies.

Tubulinosema pampeana was described associated to *B. pauloensis* in Argentina [53]. The prevalence of this parasite in queens, wild workers and laboratory workers was low, coinciding with previous results obtained in Argentina [53]. However, in previous study in our country, Plischuk *et al.* [42] found *T. pampeana* in 36.2% of the sampled *B.*

pauloensis queens and only in 1.8% of the workers, suggesting that time of the year may also influence the prevalence. Strikingly, both in Argentina and in Uruguay *T. pampeana* was only spoted in a few zones [42,53]. The impact that this new microsporidium can have at an individual or colony level is unknown. Plischuk *et al.* [53] found it infecting fat, neural and connective tissues, Malpighian tubules, muscle cells and digestive tract, so relevant effects are expected at individual level.

~~Parasites~~

The nematode *S. bombi* is a parasite widely distributed throughout the world, that has been founds in approximately 30 species of bumblebees [78]. In this study it was found in Uruguayan bumblebee queens, but at lower prevalence than found in a previous study [42]. This nematode has also been reported in the neighbor country Argentina [79]. Just like with microsporidia, the variations in the proportion of affected queens could be due to the site and time of the collection, and especially due to the conditions of hibernation. It can cause queen infertility and make them fly over the ground and for less time [58,78,80]. This nematode has a great incidence in the success of the laboratory breeding, since when present in a queen, it will not allow her to start a colony [79].

~~Parasitoids~~

Diptera larvae from Conopidae family are parasitoid with a wide dispersion, which have been heavily associated to bumblebees. Its presence can trigger abnormal responses in

bumblebees: they change their eating pattern, spend more time outside of the nest and exhibit a burial behavior during the last stages of parasitoidism [81,82]. In this study, it was found in wild workers (16.2%) and queens (10.5%). These prevalences value are superior to those found by Plischuk *et al.* (28%) [42], even though it has to be considered that in this study a lower sample size was used.

~~RNA viruses~~

Different RNA viruses (BQCV, SBV, DWV y ABPV) were detected in Uruguayan bumblebees; over 80% of the specimens exhibited at least one of them. Those viruses are frequently found in honey bees around the world [83], including Uruguay [73,76,84]. Since they have been reported to be associated to other insects, they should be considered as multi-hosts pathogens [14,22,28]. ~~BQCV virus showed the higher prevalence (80.8%), followed by SBV (17.6%), ABPV (17.6%) and DWV (6%).~~

Confinement conditions of the bumblebees during the artificial breeding do not influence in the increase of the virosis, since wild workers showed higher loads of BQCV compared to laboratory breed workers. Wild workers may be more exposed to viral infections than laboratory workers, since in the field, bumblebees could exchange viruses with honey bees, for instance, through the flowers that both species visit. In this sense, recently Alger *et al*. [23] found a higher prevalence of DWV and BQCV in bumble bees compared to neighbor honey bees. Even more they detected a bee virus in 19% of the flowers. These result shows how viruses spillover can occur between two species that share food sources. DWV is well known in honey bees, and its association with *Varroa destructor* could cause important colony losses [85,86]. Different DWV variants

have been reported in honeybees, but their presence in bumblebee species and their role in the populations needs to be addressed [86–88].

Final considerations

A priori it could be considered that the conditions of confinement of the bumblebees in artificial breeding colonies, together with the abundant food and the impossibility to fly would favor the proliferation of parasites and viruses. However, wild workers showed a higher prevalence of *N. ceranae*, mites of the genus *Kuzinia*, BQCV and SBV, and higher diversity of mites, than laboratory workers. The presence of *T. putrescentiae* on the laboratory bumblebees draws attention as its mostly associated to stored foods than to insects. An explanation to this difference is that in the field bumblebees are in contact with parasites and viruses from honey bees or other pollinators, with the flowers acting as viral and pathogens hot spots [23]. Another factor that could explain the higher presence of parasites and viruses in the wild workers is that we collected forager bees, which can be of an older age than those bumblebees extracted from laboratory colonies. The bumblebee's age was not contemplated in this study and could be relevant. For instance, in the case of honey bees the *Nosema ceranae* spore count is higher in foragers than in nurses [12].

Parasites and viruses found in laboratory workers can come from two sources: the queen or the pollen the larvae were fed with (corbicular pollen from honey bees). Bumblebee queens exhibited every parasite and virus searched in this study, with a high level of infection by *N. ceranae* (although of low prevalence) and a high diversity of

mites. This is expectable if we consider that queens are the only individuals that survive the decay of the colony and the parasites depend in good measure of them to endure in time till the start of a new colony [57].

The results of this study complement those carried out by Arbulo *et al*. [15], Plischuk *et al.* [42] and Revainera *et al*. [43], improving the sanitary map of the native bumblebees of Uruguay. Besides that, this study evidence the risk of trading practices of domesticated animals, which may act as reservoirs favoring the spread of multiple pathogens, and their spill over to native species.

~~Bibliography~~

1. Klein AM, Vaissière BE, Cane JH, Steffan-Dewenter I, Cunningham SA, Kremen C, et al. Importance of pollinators in changing landscapes for world crops. Proc R Soc B Biol Sci. 2007;274(1608):303–13.
2. Potts SG, Biesmeijer JC, Kremen C, Neumann P, Schweiger O, Kunin WE. Global pollinator declines: Trends, impacts and drivers. Trends Ecol Evol [Internet]. 2010;25(6):345–53. Available from: http://dx.doi.org/10.1016/j.tree.2010.01.007
3. Chaplin-Kramer R, Sharp RP, Weil C, Bennett EM, Pascual U, Arkema KK, et al. Global modeling of nature's contributions to people. Science (80-). 2019;366(6462):255–8.
4. Williams PH, Osborne JL. Bumblebee vulnerability and conservation world-wide. Apidologie. 2009;40(3):367–87.

5. Cameron SA, Lozier JD, Strange JP, Koch JB, Cordes N, Solter LF. Patterns of widespread decline in North American bumble bees. 2011;108(2).

6. Daszak P, Cunningham AA, Hyatt AD, Daszak P, Cunningham AA, Hyatt AD. Emerging Infectious Diseases of Wildlife- Threats to Biodiversity and Human Health. 2000;287(5452):443–9.

7. Manley R, Boots M, Wilfert L. Emerging viral disease risk to pollinating insects: Ecological, evolutionary and anthropogenic factors. J Appl Ecol. 2015;52(2):331–40.

8. Graystock P, Goulson D, Hughes WOH. Parasites in bloom: Flowers aid dispersal and transmission of pollinator parasites within and between bee species. Proc R Soc B Biol Sci. 2015;282(1813).

9. Evans E. From Humble Bee to Greenhouse Pollination Workhorse: Can We Mitigate Risks for Bumble Bees? Bee World [Internet]. 2017;94(2):34–41. Available from: https://www.tandfonline.com/doi/full/10.1080/0005772X.2017.1290892

10. Hicks BJ, Pilgrim BL, Perry E, Marshall HD. Observations of native bumble bees inside of commercial colonies of *Bombus impatiens* (Hymenoptera: Apidae) and the potential for pathogen spillover. Can Entomol. 2018;150(4):520–31.

11. Tokarev YS, Huang WF, Solter LF, Malysh JM, Becnel JJ, Vossbrinck CR. A formal redefinition of the genera *Nosema* and *Vairimorpha* (Microsporidia: *Nosematidae*) and reassignment of species based on

molecular phylogenetics. J Invertebr Pathol [Internet]. 2020;169(August 2019):107279. Available from: https://doi.org/10.1016/j.jip.2019.107279

12. Higes M, García-Palencia P, Botías C, Meana A, Martín-Hernández R. The differential development of microsporidia infecting worker honey bee (*Apis mellifera*) at increasing incubation temperature. Environ Microbiol Rep. 2010;2(6):745–8.

13. Sokolova YY, Sokolov IM, Carlton CE. Identification of *Nosema bombi* Fantham and Porter 1914 (Microsporidia) in *Bombus impatiens* and *Bombus sandersoni* from Great Smoky Mountains National Park (USA). J Invertebr Pathol [Internet]. 2010;103(1):71–3. Available from: http://dx.doi.org/10.1016/j.jip.2009.10.002

14. Li J, Chen W, Wu J, Peng W, An J, Schmid-hempel P. Diversity of *Nosema* associated with bumblebees (*Bombus* spp .) from China q. Int J Parasitol [Internet]. 2012;42(1):49–61. Available from: http://dx.doi.org/10.1016/j.ijpara.2011.10.005

15. Arbulo N, Antúnez K, Salvarrey S, Santos E, Branchiccela B, Martín-Hernández R, et al. High prevalence and infection levels of *Nosema ceranae* in bumblebees *Bombus atratus* and *Bombus bellicosus* from Uruguay. J Invertebr Pathol [Internet]. 2015;130:165–8. Available from: http://dx.doi.org/10.1016/j.jip.2015.07.018

16. Plischuk S, Lange CE. *Bombus brasiliensis* Lepeletier (Hymenoptera, Apidae) infected with *Nosema ceranae* (Microsporidia). Rev Bras Entomol [Internet]. 2016;60(4):347–51. Available from:

http://dx.doi.org/10.1016/j.rbe.2016.06.003

17. Freitas BM, Imperatriz-fonseca VL, Medina LM, De A, Peixoto M, Galetto L, et al. Diversity , threats and conservation of native bees in the Neotropics To cite this version : HAL Id : hal-00892033 Review article Diversity , threats and conservation of native bees in the Neotropics *. 2009;

18. Porrini MP, Porrini LP, Garrido PM, Melo C De, Porrini DP, Muller F, et al. *Nosema ceranae* in South American Native Stingless Bees and Social Wasp. 2017;2–5.

19. Nemésio A. Orchid bees (Hymenoptera, Apidae) of the Brazilian Atlantic Forest. Zootaxa. 2009;2041:1–242.

20. Chen YP, Siede R. Honey Bee Viruses. Adv Virus Res. 2007;70(07):33–80.

21. Chen YP, Pettis JS, Corona M, Chen WP, Li CJ, Spivak M, et al. Israeli Acute Paralysis Virus: Epidemiology, Pathogenesis and Implications for Honey Bee Health. PLoS Pathog. 2014;10(7).

22. Gamboa V, Ravoet J, Brunain M, Smagghe G, Meeus I, Figueroa J, et al. Bee pathogens found in *Bombus atratus* from Colombia: A case study. J Invertebr Pathol [Internet]. 2015;129:36–9. Available from: http://dx.doi.org/10.1016/j.jip.2015.05.013

23. Alger SA, Burnham PA, Boncristiani HF, Brody AK. RNA virus spillover from managed honeybees (*Apis mellifera*) to wild bumblebees (*Bombus* spp.). PLoS One [Internet]. 2019;14(6):e0217822. Available from:

http://dx.doi.org/10.1371/journal.pone.0217822

24. Ueira-Vieira C, Almeida LO, de Almeida FC, Amaral IMR, Brandeburgo MAM, Bonetti AM. Scientific note on the first molecular detection of the acute bee paralysis virus in Brazilian stingless bees. Apidologie. 2015;46(5):628–30.

25. Alvarez LJ, Reynaldi FJ, Ramello PJ, Garcia MLG, Sguazza GH, Abrahamovich AH, et al. Detection of honey bee viruses in Argentinian stingless bees (Hymenoptera: Apidae). Insectes Soc [Internet]. 2018;65(1):191–7. Available from: http://dx.doi.org/10.1007/s00040-017-0587-2

26. Lucia M, Reynaldi FJ, Sguazza GH, Abrahamovich AH. First detection of deformed wing virus in *Xylocopa augusti* larvae (Hymenoptera: Apidae) in Argentina. J Apic Res [Internet]. 2014;53(4):466–8. Available from: https://www.tandfonline.com/doi/full/10.3896/IBRA.1.53.4.11

27. Bailes EJ, Deutsch KR, Bagi J, Rondissone L, Brown MJF, Lewis OT. First detection of bee viruses in hoverfly (syrphid) pollinators. Biol Lett. 2018;14(2):4–7.

28. Levitt AL, Singh R, Cox-foster DL, Rajotte E, Hoover K, Ostiguy N, et al. Cross-species transmission of honey bee viruses in associated arthropods. Virus Res [Internet]. 2013;176(1–2):232–40. Available from: http://dx.doi.org/10.1016/j.virusres.2013.06.013

29. Abrahamovich AH, Tellería MC, Díaz NB. *Bombus* species and their associated flora in Argentina. Bee World. 2001;82(2):76–87.

30. Abrahamovich AH, Diaz NB, Lucia M. Identificación de las “abejas sociales” del género *Bombus* (Hymenoptera, Apidae) presentes en la Argentina: clave pictórica, diagnosis, distribución geográfica y asociaciones florales. Rev la Fac Agron La Plata. 2007;106(2):165–76.

31. Aldana J, Cure J, Almanza M, Vecil D, Rodríguez D. Efecto de *Bombus atratus* (Hymenoptera: Apidae) sobre la productividad de tomate (*Lycopersicon esculentum* Mill.) bajo invernadero en la Sabana de Bogotá, Colombia. Agron Colomb. 2007;25(1):13–4.

32. Poveda Coronel CA, Riaño Jimenez D, Aguilar Benavides L, Cure JR. Efficiency of pollination by orphan colonies of *Bombus atratus* (Hymenoptera: Apidae) in strawberry (Fragaria X Ananassa) in greenhouse. Acta Biol Colomb. 2018;23(1):73–9.

33. Salvarrey S. Characteristics of the tomato fruit (*Solanum lycopersicum*) using native bumblebees (*Bombus atratus*) as pollinators in greenhouse Características del fruto de tomate (*Solanum lycopersicum*) utilizando abejorros nativos (*Bombus atratus*) como polinizadores.

34. Salvarrey S, Arbulo N, Rossi C, Santos E, Salvarrey L, Invernizzi C. Utilización de abejorros nativos (*Bombus atratus* Franklin y *Bombus bellicosus* Smith) para mejorar la producción de semillas del trébol rojo (*Trifolium pratense*). 2017;95–104.

35. Cruz P, Escobar A, Almanza MT, Cure JR. Implementación de mejoras para la cría en cautiverio de colonias del abejorro nativo *Bombus pauloensis* (= *B. atratus*) (Hymenoptera: Apoidea). Rev Fac Ciencias

Básicas. 2017;4(1):70–83.

36. Salvarrey S, Arbulo N, Santos E, Invernizzi C. Cría artificial de abejorros nativos *Bombus atratus* y *Bombus bellicosus* (Hymenoptera, Apidae). Agrociencia Uruguay. 2013;17(2):75–82.

37. Velthuis HHW. The historical background of the domestication of the bumble-bee, Bombus terrestris, and its introduction in agriculture. Pollinat Bees - Conserv Link Between Agric Nat. 2002;177–84.

38. Velthuis HHW, Van Doorn A. A century of advances in bumblebee domestication and the economic and environmental aspects of its commercialization for pollination. Apidologie. 2006;37(4):421–51.

39. Graystock P, Yates K, Darvill B, Goulson D, Hughes WOH. Emerging dangers: Deadly effects of an emergent parasite in a new pollinator host. J Invertebr Pathol [Internet]. 2013;114(2):114–9. Available from: http://dx.doi.org/10.1016/j.jip.2013.06.005

40. Murray TE, Coffey MF, Kehoe E, Horgan F. Pathogen prevalence in commercially reared bumble bees and evidence of spillover in conspecific populations. Biol Conserv. 2013;159(January):269–76.

41. Santos E, Arbulo N, Salvarrey S, Invernizzi C. Distribución de las especies del género Bombus Latreille (Hymenoptera, Apidae) en Uruguay. Rev la Soc Entomológica Argentina [Internet]. 2017;76(1–2):22–7. Available from: https://doi.org/10.25085/rsea.761203

42. Plischuk S, Salvarrey S, Arbulo N, Santos E, Skevington JH, Kelso S, et al. Pathogens, parasites, and parasitoids associated with bumble bees

(*Bombus* spp.) from Uruguay. Apidologie. 2017;48(3):298–310.

43. Revainera PD, Salvarrey S, Santos E, Arbulo N, Invernizzi C, Plischuk S, et al. Phoretic mites associated to *Bombus pauloensis* and *Bombus bellicosus* (Hymenoptera: Apidae) from Uruguay. J Apic Res [Internet]. 2019;58(3):455–62. Available from: https://doi.org/10.1080/00218839.2018.1521775

44. Arbetman MP, Meeus I, Morales CL, Aizen MA. Alien parasite hitchhikes to Patagonia on invasive bumblebee. Biol Invasions. 2013;(March).

45. Morales CL, Arbetman MP, Cameron SA, Aizen MA, Morales CL, Arbetman MP, et al. Rapid ecological replacement of a native bumble bee by invasive species. Front Ecol Environ. 2013;

46. Aizen MA, Smith-Ramírez C, Morales CL, Vieli L, Sáez A, Barahona-Segovia RM, et al. Coordinated species importation policies are needed to reduce serious invasions globally: The case of alien bumblebees in South America. J Appl Ecol [Internet]. 2018;(March). Available from: http://doi.wiley.com/10.1111/1365-2664.13121

47. Schmid-Hempel R, Eckhardt M, Goulson D, Heinzmann D, Lange C, Plischuk S, et al. The invasion of southern South America by imported bumblebees and associated parasites. J Anim Ecol. 2014;83(4):823–37.

48. Hunter PE. The genus *Pneumolaelaps* with description of three new species (Acarina: Laelaptidae). J Kansas Entomol Soc. 1966;39:357–69.

49. Hunter PE, Husband RW. *Pneumolaelaps* (Acarina: Laelapidae) mites from North America and Greenland. Florida Entomol. 1973;59:77–91.

50. Putatunda BN, Aggarwal K, Kapil RP. Two new species of Kuzinia (Acarina: Acaridae) associated with bees (Hymenoptera) from India. Indian J Acarol. 1983;8(2):57–62.

51. Krantz GW, Walter DE. A manual of acarology. 2009.

52. Solter LF, Becnel JJ, Oi DH. Microsporidian entomopathogens. San Diego. In: F. E. Vega & H. K. Kaya (eds.), editor. In Insect Pathology and Microbial Pest Control. Second Edi. San Diego; 2012.

53. Plischuk S, Sanscrainte ND, Becnel JJ, Estep AS, Lange CE. a pathogen of the South American bumble bee *Bombus atratus*. J Invertebr Pathol [Internet]. 2015;126:31–42. Available from: http://dx.doi.org/10.1016/j.jip.2015.01.006

54. Undeen HH, Vávra J. Research methods for entomopathogenic protozoa. In: Lacey L, editor. Manual of techniques in insect pathology. New York; 1997. p. 117–51.

55. Pfaffl W. A new mathematical model for relative quantification in real-time RT–PCR. Nucleic Acids Res. 2001;425(3):2002–7.

56. Binns ES. Phoresy as migration-some functional aspects of phoresy in mites. Biol Rev [Internet]. 1982;57(4):571–620. Available from: https://doi.org/10.1111/j.1469-185X.1982.tb00374

57. Huck K, Schwarz HH, Schmid-Hempel P. Host choice in the phoretic mite *Parasitellus fucorum* (Mesostigmata: Parasitidae): Which bumblebee caste is the best? Oecologia. 1998;115(3):385–90.

58. Goulson D. Bumblebees: behaviour, ecology and conservation. Second

Edi. Goulson D, editor. New York; 2010. 317 p.

59. Maggi MD, Lucia M, Abrahamovich AH. Study of the acarofauna of native bumblebee species (*Bombus*) from Argentina. Apidologie. 2011;42:280–92.

60. Rozej E, Witaliński W, Szentgyörgyi H, Wantuch M, Moroń D, Woyciechowski M. Mite species inhabiting commercial bumblebee (*Bombus terrestris*) nests in Polish greenhouses. Exp Appl Acarol. 2012;56(3):271–82.

61. Revainera P, Lucia M, Abrahamovich AH, Maggi M. Spatial aggregation of phoretic mites on *Bombus atratus* and *Bombus opifex* (Hymenoptera: Apidae) in Argentina. Apidologie. 2014;45(5):579–89.

62. Revainera PD, Quintana S, Fernández de Landa G, Meroi Arcerito F, Lucía M, Abrahamovich AH, et al. Phoretic mites on South American bumblebees (*Bombus* spp.) as parasite carriers: a historical input. Apidologie. 2020;

63. Hubert J, Stejskal V, Kubátová A, Munzbergová Z, Váňová M, Žd'árková E. Mites as Selective Fungal Carriers in Stored Grain Habitats. Exp Appl Acarol [Internet]. 2003;29:69–87. Available from: https://doi.org/10.1023/A:1024271107703

64. Schmid-Hempel P, Schmid-Hempel R. Endoparasitic flies, pollen-collection by bumblebees and potential host parasite conflict. Oecologia. 1991;87:222–32.

65. Schwarz HH, Huck K. Phoretic mites use flowers to transfer between foraging bumblebees. Insectes Soc. 1997;44(4):303–10.

66. Goulson D. Impacts of non-native bumblebees in Western Europe and North America. Appl Entomol Zool. 2010;45(1):7–12.

67. Kissinger CN, Cameron SA, Thorp RW, White B, Solter LF. Survey of bumble bee (*Bombus*) pathogens and parasites in Illinois and selected areas of northern California and southern Oregon. J Invertebr Pathol [Internet]. 2011;107(3):220–4. Available from: http://dx.doi.org/10.1016/j.jip.2011.04.008

68. Delfinado M., Baker E. Notes on Hypopi (Acarina) Associated with Bees and Wasps (Hymenoptera). J New York Entomol Soc [Internet]. 1976;84(2):76–90. Available from: https://www.jstor.org/stable/25008994

69. Royce L, Krantz GW. Observations on pollen processing by *Pneumolaelaps longanalis* (Acari: Laelapidae), a mite associate of bumblebees. Exp Appl Acarol. 1989;7(2):161–5.

70. Fries I. *Nosema ceranae* in European honey bees (*Apis mellifera*). J Invertebr Pathol [Internet]. 2010;103(SUPPL. 1):S73–9. Available from: http://dx.doi.org/10.1016/j.jip.2009.06.017

71. Colla SR, Otterstatter MC, Gegear RJ, Thomson JD. Plight of the bumble bee: Pathogen spillover from commercial to wild populations. Biol Conserv. 2006;129(4):461–7.

72. Hicks BJ, Pilgrim BL, Perry E, Marshall HD. Observations of native bumble bees inside of commercial colonies of *Bombus impatiens* (Hymenoptera: Apidae) and the potential for pathogen spillover. Can Entomol. 2018;150(4):520–31.

73. Anido M, Branchiccela B, Castelli L, Harriet J, Campá J, Zunino P, et al. Prevalence and distribution of honeybee pathogens in Uruguay. J Apic Res. 2015;54(5):532–40.

74. Antúnez K, Anido M, Branchiccela B, Harriet J, Campa J, Invernizzi C, et al. Seasonal Variation of Honeybee Pathogens and its Association with Pollen Diversity in Uruguay. Microb Ecol. 2015;70(2):522–33.

75. Invernizzi C, Santos E, García E, Daners G, Di Landro R, Saadoun A, et al. Sanitary and nutritional characterization of honeybee colonies in *Eucalyptus grandis* plantations. Arch Zootec. 2011;60(232):1303–14.

76. Branchiccela B, Castelli L, Corona M, Díaz-Cetti S, Invernizzi C, Martínez de la Escalera G, et al. Impact of nutritional stress on the honeybee colony health. Sci Rep. 2019;9(1):1–11.

77. Rotheray EL, Osborne JL, Goulson D. Quantifying the food requirements and effects of food stress on bumble bee colony development. J Apic Res [Internet]. 2017;56(3):288–99. Available from: http://dx.doi.org/10.1080/00218839.2017.1307712

78. Poinar GO, Van der Laan PA. Morphology and life history of Sphaerularia bombi.pdf. Nematologica. 1972;18:239–52.

79. Plischuk S, Lange CE. Sphaerularia bombi (Nematoda: Sphaerulariidae) parasitizing *Bombus atratus* (Hymenoptera: Apidae) in southern South America. Parasitol Res. 2012;111(2):947–50.

80. Jones CM, Brown MJF. Parasites and genetic diversity in an invasive bumblebee. J Anim Ecol. 2014;83(6):1428–40.

81. Müller CB, Schmid-Hempel P. Exploitation of cold temperature as defence against parasitoids in bumblebees. Nature [Internet]. 1993;363:65–6. Available from: https://doi.org/10.1038/363065a0

82. Müller CB. Parasitoid induced digging behaviour in bumblebee workers. Anim Behav. 1994;48(4):961–6.

83. Bee WH, Beaurepaire A, Piot N, Doublet V, Antunez K. Diversity and Global Distribution of Viruses of the. 2020;1–25.

84. Antúnez K, D'Alessandro B, Corbella E, Ramallo G, Zunino P. Honeybee viruses in Uruguay. J Invertebr Pathol [Internet]. 2006 Sep;93(1):67–70. Available from: https://linkinghub.elsevier.com/retrieve/pii/S002220110600108X

85. de Miranda JR, Genersch E. Deformed wing virus. J Invertebr Pathol [Internet]. 2010;103(SUPPL. 1):S48–61. Available from: http://dx.doi.org/10.1016/j.jip.2009.06.012

86. Martin SJ, Highfield AC, Brettell L, Villalobos EM, Budge GE, Powell M, et al. Global Honey Bee Viral Landscape Altered by a Parasitic Mite. Science (80-). 2012;336:1304–6.

87. Dalmon A, Desbiez C, Coulon M, Thomasson M, Le Conte Y, Alaux C, et al. Evidence for positive selection and recombination hotspots in Deformed wing virus (DWV). Sci Rep [Internet]. 2017;7(January):1–12. Available from: http://dx.doi.org/10.1038/srep41045

88. Natsopoulou ME, McMahon DP, Doublet V, Frey E, Rosenkranz P, Paxton RJ. The virulent, emerging genotype B of Deformed wing virus is closely

linked to overwinter honeybee worker loss. Sci Rep. 2017;7(1):1–9.

 Click here to access/download;Figure;Fig 1.jpg

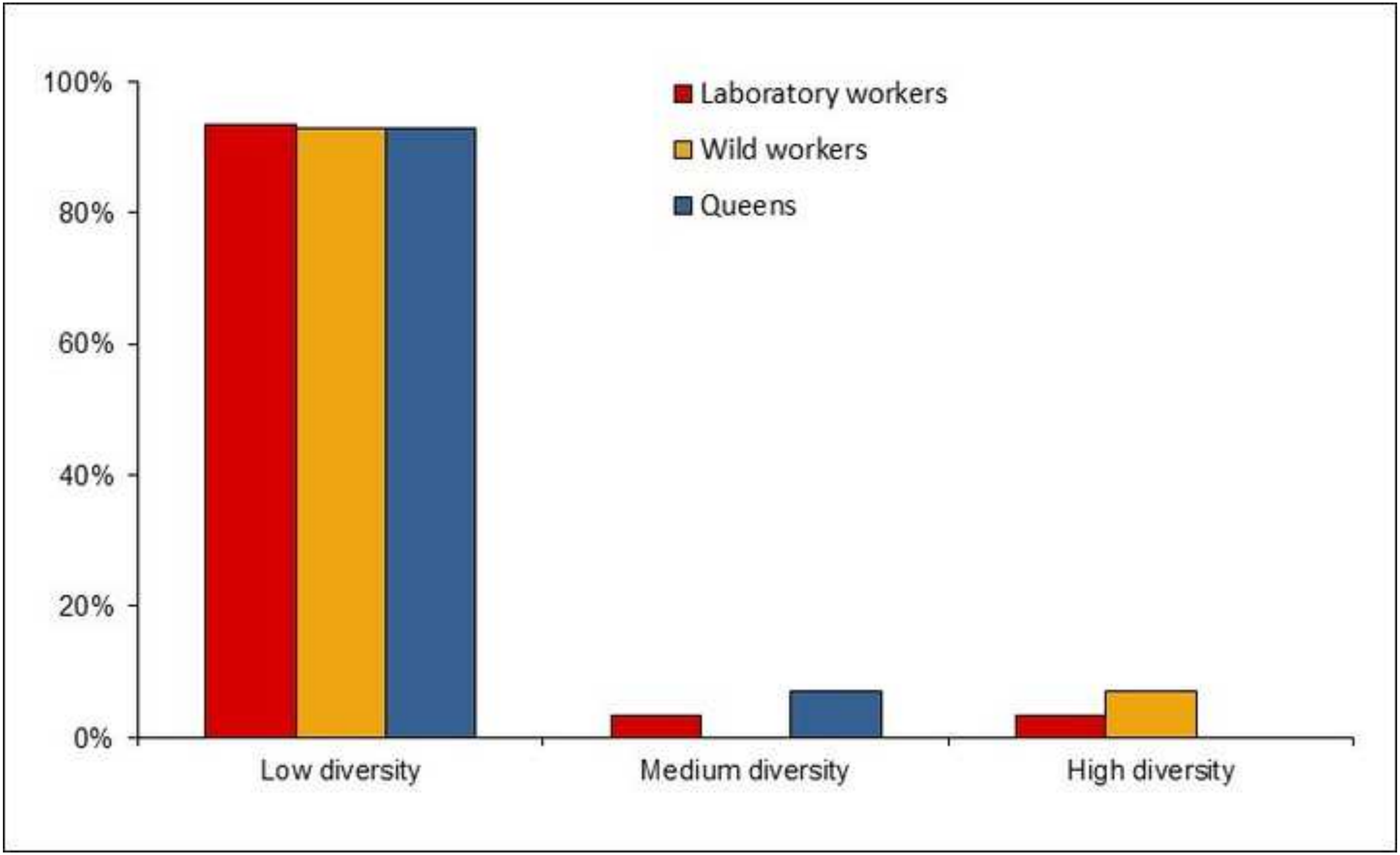

 Click here to access/download;Figure;Fig 2.jpg

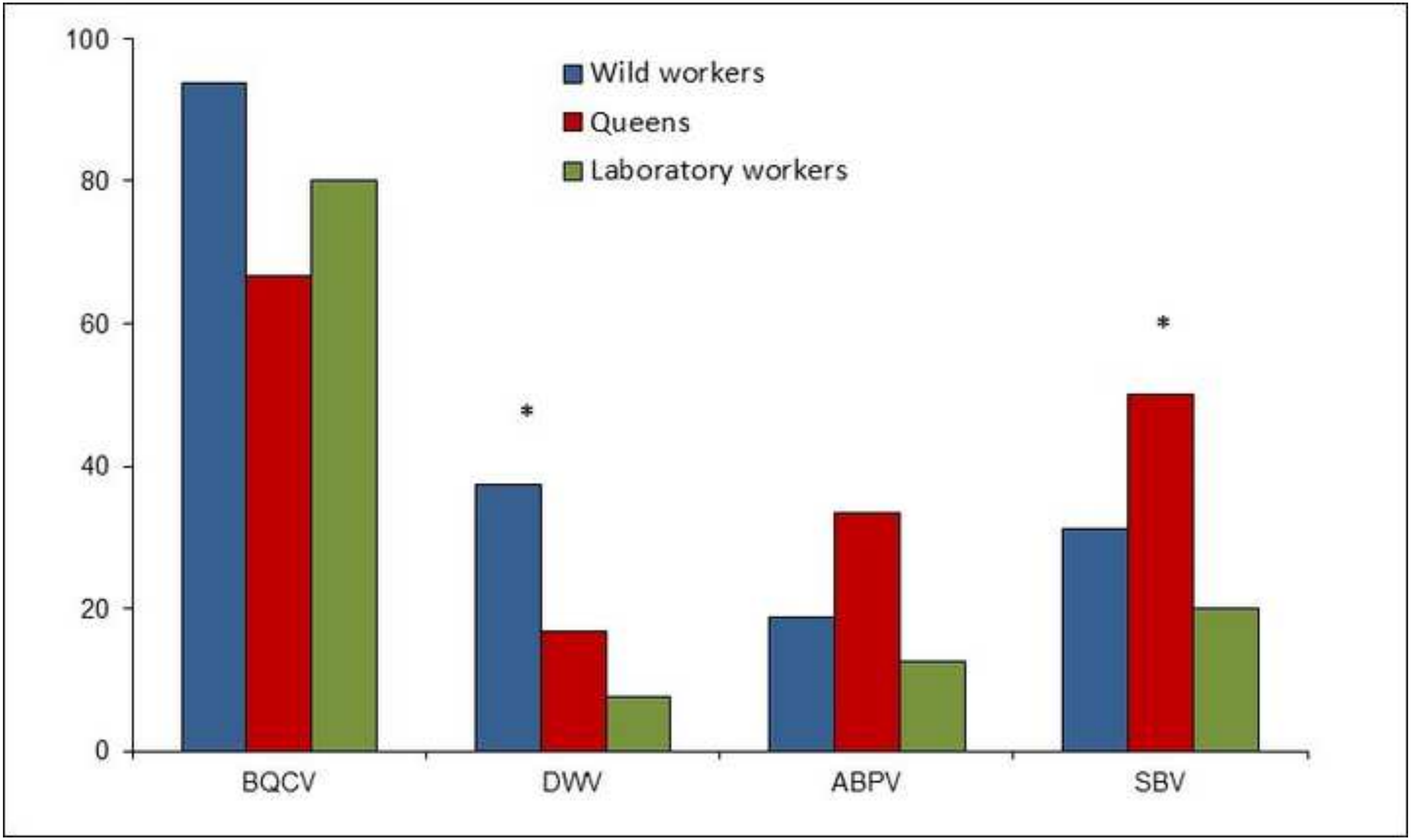

Supplement: Attachment — Submitted filename: PONE-D-20-36603_reviewer Parasites and RNA viruses in wild and laboratory reared bumblebees Bombus.pdf [file pone.0249842.s004.pdf]
