# Supplementary material for: Parasites and RNA viruses in wild and laboratory reared bumble bees Bombus pauloensis (Hymenoptera: Apidae) from Uruguay
Source: PLoS One. 2021 Apr 26;16(4):e0249842. doi: 10.1371/journal.pone.0249842 (PMC8075198; doi:10.1371/journal.pone.0249842)
Supplement: Attachment — Submitted filename: Comments to editor Plos One 13 ene 2021.docx [file pone.0249842.s003.docx]

January 13, 2021

Dear editor

The manuscript entitled " Parasites and RNA viruses in wild and laboratory reared bumblebees *Bombus pauloensis* (Hymenoptera: Apidae) from Uruguay". This study evaluated the presence of the main internal and external parasites, as well as, the RNA viruses on queens wild and laboratory workers of *B. pauloensis.* The results of the research carried out by the authors are valuable, new, and deserve to be published. However, they need improve some points, especially the Materials y Methods section, but also, Results section. I believe that some section should be reformulated and new information must be included by the authors in order to achieve a more consistent, organized article that provides relevant and novel information to the readers.

Many of the specific comments to improve this manuscript were made directly in the pdf file. Please see the pdf file.
