# Supplementary material for: Parasites and RNA viruses in wild and laboratory reared bumble bees Bombus pauloensis (Hymenoptera: Apidae) from Uruguay
Source: PLoS One. 2021 Apr 26;16(4):e0249842. doi: 10.1371/journal.pone.0249842 (PMC8075198; doi:10.1371/journal.pone.0249842)
Supplement: Attachment — Submitted filename: Response to Reviewers.doc [file pone.0249842.s005.doc]

**Response to reviewers**

Guy Smagghe, PhD
Academic Editor
PLOS ONE

I am sending a revised version of the article entitled "Parasites and RNA viruses in wild and laboratory reared bumble bees *Bombus pauloensis* (Hymenoptera: Apidae) from Uruguay" to be evaluated for its publication in PLOS ONE.

We think that suggestions made by both reviewers to the first version of the manuscript were very valuable and most of them were taken into account in the revised version.

The authors want to thank you and the reviewers for all the contributions made to improve the quality of the manuscript.

Because of a misunderstanding, we didn’t include in the submission form the information about financial disclosure statement. We detail it here hoping you can include it now:

Funding was provided by a PhD scholarship grant to S.S. from Comisión Académica de Posgrado (CAP) of the Universidad de la República (Udelar) and the Agencia Nacional de Investigación e Innovación (ANII)/register number: POSNAC_2014_1_102699. The funders had no role in study design, data collection and analysis, decision to publish, or preparation of the manuscript.

Below we include the responses to comments and suggestions from the reviewers.

Kind regards

Sheena Salvarrey

**Reviewer #1**:

The heterogeneity of the types of pathogens screened for is good, but I do miss some additional analysis. For example, the choice of only microscopic screening of internal pathogens does not make the methods very sensitive. Only two microsporidian species were chosen to screen for. Why the choice of these parasites? Why not other internal parasites like *Crithidia* sp. or *Apicystis* sp.? This might give a lot more interpretable data, and might give enough parasite diversity data to actually state some objectives and get some more information regarding parasites in bumblebees, rather than only report the findings.

**R:** We used the microscopical screening to search for the presence of several parasites that can be identified with this method (e.g. Kinetoplastidea, Neogregarinorida and Microsporidia). The presence of *Crithidia* sp. and *Apycistis* sp. was also evaluated but those parasites were not found. This information was included in the new version of the manuscript.

… I also wonder how did you distinguish between *Nosema* species? How did you see a difference between *N. ceranae* and *N. bombi* without molecular confirmation?

**R:** We are convinced that the spores were *N. ceranae* since I) previous studies carried out by molecular methods have never found *N. bombi* in *Bombus* spp. in Uruguay. Closest reports were 1,400 km far from the country (e.g.: Plischuk, 2013; Schmid-Hempel et al., 2014; Arbulo et al. 2015; Plischuk & Lange, 2016; Plischuk et al., 2017a, b), and II) careful dissections of all bees showed that spores were always infecting the gut (target tissue of *N. ceranae*) and they never were found into Malpighian tubules (target tissue of *N. bombi*).

For me the sampling set up is not very clear: why take queens from wild and rear them, and then compare them with workers from the wild but from another year? What was the original motive to do this?

**R:** We collected queens from the wild in spring 2014 (September in south hemisphere) and some of them were used for the parasites and viral analysis and others for the rearing in laboratory. Wild workers were collected in autumn (March 2015) since it is the only time of the year when they are found in great numbers in the wild, in accordance with their biological cycle. It is not possible to find many wild workers in spring. Spring 2014 and autumn 2015 belong to the same “biological year” for this species. We compared the workers reared in the lab with the workers and queens collected in the wild.

having a similar prevalence between queens and laboratory workers is not so strange, since they don’t come into contact anymore with the environment, the lab workers can only get parasites from the queens; the queens already infested will transmit their parasites, while the queens not infested cannot transmit parasites, so the prevalence will stay similar. What would be a relevant analysis, is to compare the parasite diversity between the queens and the lab workers; are there any parasites that are not present (or way less) in the lab workers but were omnipresent in the queens? For example, from your data, there doesn’t seem to be a bottle neck for *Nosema ceranae*, but a small bottleneck for *T. pampeana*. Even better would be to also analyse the parasites of the queens used for rearing, if they were kept frozen after the experiment.

**R:** We agree with your comments and we considered that some of those ideas are already included in the manuscript. As the example that you mentioned about the two microsporidia in the discussion section we discuss about other examples such as a *P. longanalis*, and SBV (Sacbrood virus). On the other hand, unfortunately the queens used for rearing were not kept frozen since laboratory colonies were used for pollination experiments; we are aware that including them in the analysis would have enrich this study.

[255] You mention mites being a possible vector, but you don’t analyses this in your data; for this you don’t necessarily have to screen the mites themselves, but you could model a relation between presence of certain mite species and prevalence/abundance/load of viruses and pathogens.

**R:** Its very interesting what you mention about modeling but unfortunately, we don’t have the necessary data (e.g. load) for all groups of pathogens and viruses to do that.

Some smaller comments: The general structure is not good yet. Some examples:
55 strange sentence ‘another example of pathogen spillover are RNA viruses’. Then you explain how viruses found in honeybees are also found in other bee species, but you don’t provide any context about why these viruses are considered to be a spillover risk.

**R:** We modified the text and your suggestions were included.

45 ‘among others’ fits better than ‘between others’.

**R:** The text was modified taking into account this observation.

Also, maybe it is good to mention multiplicative effects, like pesticide intoxication that increases pathogen susceptibility.

**R:** It was mentioned in the revised version.

85-87 I do not understand this sentence. This sentence seems to state that *B.* *terrestris* and .*B ruderatus* is present in Uruguay, while on line 72 you say there are only *B. pauloensis* and *B. bellicosus*. Also the second part of the sentence on 86 does not make sense.

**R:** The text was modified taking into account these observations.

94 you mention 33 queens, and then explain how many are used for different analysis; then you mention 40 (of these 33??) were used to start laboratory rearing. I don’t think that is physically possible… Also were the queens collected from a lab experiment or were they actually caught during foraging from the wild? This is not explained clearly.

**R:** We corrected the numbers of the total collected queens and added details to the material and methods section to improve the clarity.

There are still a lot of grammar and spelling mistakes in the text. Some examples:
84 ‘significant’ damage

107 grammatical mistake

287-289 strange sentence

327-spotted
392-strange sentence

397 strange sentence and weird conclusion?

**R:** The manuscript was revised and improved, taking into account all the suggestions.

**Reviewer 2**

**Line 2-** Bumble bees

**R:** It was corrected.

**Line 20-** Please change bumblebees by bumble bee. I recommend making this change in the full text. Also in the case of honeybees by honey bees.

**R:** It was corrected.

**Line 35-** bumble bees

**R:** It was corrected.

**Line 45-** Please, to continue in the Line 45. ...between others (2). In particular, ....

**R:** It was corrected.

**Line 49-** Sentence in **Line**s 49-51, tend to ensure that *Apis mellifera* is main agent of dispersion of pathogens to other insect species, which is not clear at all. Please reconsider the setence. I recomend to consider the honey bees as one of posible agent of pathogen dispersion. ....while others have a broad host spectrum [6,7]. There is e.g. that the extended commerce and movement of managment bees such as honey bees, has led ....

**R**: We agree that *A. mellifera* is not the only agent of pathogens dispersion but that is not the intention of the reference sentence. We just wanted to highlight the role of commercial colonies as pathogens and parasites reservoirs basing this idea on several references (6,8,9,10,11). We made little modification in the text to clarify the idea, including bumble bees among the commercial colonies.

**Line 54-** Bumble bees

**R:** It was corrected.

**Line 50-** *Apis mellifera* add “L.”

**R:** It was corrected.

**Line 52-** *Nosema ceranae* add “Fries”

**R:** It was corrected.

**Line 62-** Friese

**R:** It was corrected.

**Line 67-** Move to **Line** 88.

From a sanitary point of view, the artificial breeding conditions (high density of individuals, impossibility of going out to defecate and forage, and limited food availability), can increase the survival and multiplication of different pathogens, facilitating the proliferation and transmission of diseases [39,40]. Thus, the aim the this study was perform an exhaustive survey...

**R:** The text was modified as suggested.

**Line 72-** Smith

**R:** It was corrected.

**Line** **75**- To include species descriptor

**R:** It was corrected.

**Line** **76**- Dufour

**R:** It was corrected.

**Line** **77**- To include Descriptor of these species

**R:** It was corrected.

**Line** **81**- L. and F.

**R:** It was corrected.

**Line** **82**- Guérin-Méneville

**R:** It was corrected.

**Line** **85**- I recommend to read and to include Arismendi et al 2021. Occurrence of bee viruses and pathogens associated with emerging infectious diseases in native and non-native bumble bees in southern Chile. Biological Invasion <https://doi.org/10.1007/s10530-020-02428-w>

**R:** The paper was cited and included in the references (47).

**Line** 96- Why did not you use all the samples for the viral, ectoparasites and other pathogens analysis? That is, the 33 samples. I believe that these pathogens/parasites can be detected in one sample.

How did you maintain the samples? in ethanol? under cold condition (-20°C/-80°C). Please explain/answer in details these questions

**R:** It was not possible to perform all analysis in the same individuals since every parasite/ pathogen requires different conditions of sample storage (-20 or -80°C) and different methods for sample processing. For example, samples for viral analysis requires storage at -80°C and the complete specimen is homogenized for RNA extraction. On the other hand, samples for internal parasites analysis require storage at -20°C, specimens must be dissected, the midgut extracted and observed. Procedures are not compatible to be performed in the same individual.

**Line** 97- There is not clear!

How many queens were collected after hibernation period? 33 or 73?.

I feel that, there were collected 73 queens (see Lines 94-97). In which case, 33 were used for pathogen/parasites analisys and 40 queens were used to start rearing under laboratory condiction.

Please clarify!

**R:** In total 73 queens were collected, 33 for analysis and 40 for rearing. It was clarified in the text.

**Line** **99**- Similar to above comments, why not use the 92 worker bumble bees for internal and external pathogen/parasites?

**R**: As explained before, it was not possible to perform all analysis in the same individuals since every parasite/ pathogen requires different conditions of sample storage (-20 or -80°C) and different methods for sample processing.

**Line** **113**- to continue in the **Line** 112.

**R:** It was corrected.

**Line** **138**- No italic

**R:** It was corrected.

**Line** **139**- No italic

**R:** It was corrected.

**Line** **141**-add dut

**R:** It was corrected.

**Line** **150**- No italic

**R:** It was corrected.

**Line** **151**- ).

**R:** It was corrected.

**Line** **152**- In the Table 1, only β-actin was listed. Did you also used RPS5?

**R:** Only β-actin was used. It was corrected in the text.

**Line** **160**- Please indicate the statistical software.

**R:** InfoStat software was used. It was included in the text.

**Line** **172**- Please add real p value. In this case must be report as: Chi-square: = 7.52; p =0.006; df = 1).

**R:** It was corrected

**Line** **174**- Is it the real p-value? I believe that the p-value is p <001. Please clarify!

**R:** It was corrected

**Table 2-** Please indicate N° samples

Indicate N° samples

Where is the wild queens values?

You stated in the

**Line**s 96-97 that 33 queens were collected for pathogens/parasites analysis

**R:** The text was modified as suggested.

**Line** **180**- p = 0003

**R:** The real p value was added

**Line** **182**- co-infestation

**R:** It was corrected.

**Line 187**- Please to include title in the Y-axis in the Figure 1, anexed in this manuscript.

**R:** It was corrected.

**Line 191-** Delete **Line**s 191-192. To include this information at end of Figure 1.

**R:** We think it should be explained in the figure legend to understand the figure.

**Line 198**-real p-value

**R**: It was included.

**Line 199**-real p-value

**R:** It was included.

**Line 202, 203, 207, 208-** change “individual” for **“**bee”

**R:** It was corrected.

**Line 220-** Real p-value

**R:** It was included.

**Line 223-** I recommend to include ct values in supplementary material

**R:** If the reviewer think it is essential, we can include it. However, we think it does not provide substantial information to the manuscript.

**Line 227-** Real p-value

**R:** It was included.

**Line 228-** Real p-value

**R:** It was included.

**Line 229-** Please to include title in Y-axis

**R:** It was included.

Please clarify the asteristic in the bars. Who or what is it being compared to? You must to be illustrative and clear to the reader.

**R:** It was corrected

**Line 232-** Move this setence at end of Figure 2.

**R:** We think this information should be in the figure legend.

spell asterisk. No symbol.

The asterisk indicates ...

**R**: It was corrected.

**Line** **235-238**- There were not shown the viral load.

I am confused. If you used Real Time PCR, there exist the posibility to make a viral quantification. Futhermore, you cited Pfaffl 2001.

Pfaffl W. A new mathematical model for relative quantification in real-time 590 RT–PCR. Nucleic Acids Res. 2001;425(3):2002–7.

If you did not quantified the viral load in the infected bumble bees, I recommend to do it. I believe this information could be usefull to related the viral prevalence with viral load. One thing is prevalence (positive cases) and the other, is the infection level (high, medium or low load of viral infection). There exist the posibility that hight prevalence could be related to high viral load. On the hand, low prevalence, could be associated to low viral titer. The viral load could have implicance in viral dispersion between bee species.

Of course that the viral prevalence is importante index, however, without viral load, it is difficult to conceptualize the intensity of infection or exposure of infected bees. This provides important information on if bee viruses are present in bumble bees at levels likely to have consequences for their health.

For more details, please see:

Dolezal AG, Hendrix SD, Scavo NA, Carrillo-Tripp J, Harris MA, Wheelock MJ, et al. (2016) Honey Bee Viruses in Wild Bees: Viral Prevalence, Loads, and Experimental Inoculation. PLoS ONE 11(11): e0166190. doi:10.1371/journal. pone.0166190

Alger SA, Burnham PA, Boncristiani HF, Brody AK (2019) RNA virus spillover from managed honeybees (*Apis mellifera*) to wild bumblebees (*Bombus* spp.). PLoS ONE 14(6): e0217822. https://doi.org/10.1371/journal.

pone.0217822

Alger SA, Burnham PA, Brody AK (2019) Flowers as viral hot spots: Honey bees (*Apis mellifera*) unevenly deposit viruses across plant species. PLoS ONE 14(9): e0221800.

https://doi. org/10.1371/journal.pone.0221800

Arismendi et al 2021. Occurrence of bee viruses and pathogens associated with emerging infectious diseases in native and non-native bumble bees in southern Chile. Biological Invasion <https://doi.org/10.1007/s10530-020-02428-w>

**R:** β-actin mRNA was amplified in each sample as a control of correct RNA manipulation and extraction. The text was modified accordingly. The reference Pfaffl 2001 was not adequate, since viral quantification was not carried out.

We agree that absolute quantification would provide interesting information and will allow the comparison between viral levels in honey bees and bumble bees. However, at this point it is not possible to perform since we did not include adequate standard curves (dilution series of cloned PCR products or commercial gene fragments, for each virus).

In spite of that, we think that information regarding RNA viruses prevalence, together with the information of other parasites and pathogens provides useful information.

**Line** **244**- This is the objective. It should be in Lines 88-90.

**R:** It was corrected.

**Line** **246**- Delete. It does not provide relevant information.

**R:** It was deleted.

**Line** **249**- I recommend to delete the subtitles in Discussion section

**R:** Subtitles were deleted.

**Line** **250**- ... phoretic mites in bumble bee... There are tracheal mites which to be harmful for bumblebees.

**R:** It was corrected.

**Line** **254**- and

**R:** It was added

**Line** **273**- Please to include reference!

**R:** It was included.

**Line** **275**- To include reference

**R:** It was included.

66 - Koulianos, S., Schwarz, H.H. (1999) Reproduction, development and diet of *Parasitellus* *fucorum* (Mesostigmata: Parasitidae), a mite associated with bumblebees (Hymenoptera: Apidae). J. Zool., 248, 267–269.

67- Royce, L.A., Krantz, G.W. (1989) Observations on pollen processing by *Pneumolaelaps* *longanalis* (Acari, Laelapidae), a mite associate of bumblebees. Exp. App. Acarol., 7, 161–165.

**Line** **287**- To include comma (,)

**R:** It was corrected.

**Line** **298**- Delete

**R:** It was deleted.

**Line** **301**- ...Apis cerana (70). However,

**R:** It was corrected

**Line** **310**- may or could?

**R:** It was modified as suggested.

**Line** **316**- Even more,

**R:** It was modified as suggested.

**Line** **317**- honey bee

**R:** It was modified as suggested.

**Line 324**- Uruguay

**R:** It was modified as suggested.

**Line** **332**- Delete

**R**: It was modified as suggested.

**Line** **344**- Delete

**R**: It was modified as suggested.

**Line** **353**- Delete

**R**: It was modified as suggested.

**Line** **358**- These data are results.

**R:** It was modified as suggested.

**Line** **361**- Viral load data were not shown in the manuscript.

R: Viral load was not estimated. The text was modified to viral prevalence.

**Line** **378**- no italic

**R:** It was corrected.

**Line** **400**- Add Acknowledgments and Author Contributions

**R:** It was included according to journal guidelines

**Line** **401**- References

**R**: It was corrected.
